# Establishing Baseline Radiological Metrics of the Talus and Calcaneus: A Retrospective Analysis of Computed Tomography Imaging in an Irish Cohort

**DOI:** 10.7759/cureus.48118

**Published:** 2023-11-01

**Authors:** David O'Sullivan, Martin Davey, Diane Bergin, SR Kearns

**Affiliations:** 1 Department of Trauma and Orthopaedics, Galway University Hospitals, Galway, IRL; 2 Trauma and Orthopaedics, Royal College of Surgeons, Dublin, IRL; 3 Department of Radiology, Galway University Hospitals, Galway, IRL; 4 Orthopaedics, University Hospital Galway, Galway, IRL

**Keywords:** foot anatomy, subtalar joint, measurements, ct (computed tomography) imaging, ankle and foot

## Abstract

Introduction

The subtalar joint anatomy is complex and heterogeneity in its morphology creates unique challenges for foot and ankle surgeons. Anatomical metrics used for prosthesis design are well established. However, there is a paucity of literature quantifying foot and ankle measurement techniques and metrics used for prosthesis design. The aim of this study was to document reproducible measurement techniques and quantify talar and calcaneal metrics in a sample of Irish patients on computed tomography imaging to aid in the design of a novel hindfoot plate.

Methods

A retrospective analysis of consecutive foot and ankle computed tomography images performed at our institution was undertaken. Five measurements were performed on each foot and ankle image. Statistical analysis was performed to identify if a correlation existed between measurements.

Results

Sixty-four CTs met the inclusion criteria. Talar body height 27.1 mm (SD 2.17 mm), talar neck width 32.7 mm (3.16 mm), talar head height 25.41 (SD 2.16 mm), lateral process to posterior talus 23.6 (2.64 mm), calcaneal height 43.8 mm (SD 3.9 mm). A positive correlation was identified between all measurements.

Conclusion

This study identified that there was a low degree of heterogeneity in talar and calcaneal measurements in an Irish cohort. Furthermore, the metrics used in this study will provide valuable information for the preliminary design of a novel hindfoot plate.

## Introduction

The anatomy of the subtalar joint is complex and discrepancies in opinion persist with respect to its exact description in anatomical literature [1-3]. Previous literature supports this, with large variability reported in talar and subtalar joint morphology existing at a population level [2-4]. Anatomical variability of the hindfoot combined with intra-operative technical challenges such as trans-articular screw alignment has contributed to isolated subtalar fusion surgery demonstrating non-union rates as high as 12%-24% [5,6].

Anatomical metrics for prosthetic design in orthopedic surgery can be obtained through a number of different methods including cadaveric studies and radiological analysis [7]. computed tomography imaging (CT) is a reproducible, inexpensive investigation that has been used previously when obtaining metrics prosthesis design, and it is widely accepted that CT imaging is the imaging modality of choice in the evaluation of bony injuries in the foot and ankle [8,9]. Previously published international literature has highlighted that variability exists in ankle metrics depending on a variety of factors, including ethnic origin and sex [4,10].

The lead investigators of this project identified a paucity of established measurement techniques for the hindfoot. Concurrently, there is a lack of published data on foot and ankle measurements using CT as an imaging modality. Furthermore, the lead investigators have hypothesized that a novel talocalcaneal plate design may better guide alignment for trans-articular screw placement, and decrease postoperative non-union rates in patients undergoing subtalar fusion surgery. In order to design a construct suitable for preclinical computer-aided design and biomechanical analysis, preliminary measurements of the talus and calcaneus are warranted. Therefore, the aim of this study was to quantify talar and calcaneal metrics in a sample of Irish patients using CT imaging to aid in the design of a novel plate for sub-talar arthrodesis.

## Materials and methods

Ethical approval was acquired from our institution. Consecutive patients who underwent a CT of the foot and ankle in our institution between June 2020 and April 2021 were screened for inclusion. Studies were included if they met the following criteria: (1) the patient was skeletally mature, (2) the study included osseous structures of the entire midfoot, hind foot, tibia, and fibula, and (3) were of adequate quality (as outlined below). The exclusion criteria included: (1) CT scans of patients under the age of 18, (2) the presence of talus or calcaneal fractures, (3) previous talar or calcaneal surgery, (4) foot and ankle deformities such as Charcot foot, or (5) co-existing talar osteophytes.

CT imaging

CT imaging was performed in a tertiary referral center comprising three CT scanners. Imaging was performed using a Siemens SOMATON Definition 128 configuration and two Siemens SOMATON sensation 64 configuration scanners. CT sequences performed were of slice thickness 1 mm, kernel B70s with bone and soft tissue algorithms. Images were stored on the Picture Archive and Communication System (PACS).

The CT images were exported to imaging software (IMPAX Agfa- Enterprise Gevaert, Mortsel, Belgium) for analysis. Multi-planar reconstruction (MPR) was used to align images on the correct anatomical axis to each plane before measurements were recorded.

Measurements

Sagittal, coronal, and axial plane alignment of the talus and calcaneus in anatomical orientation was essential for accuracy and reproducibility in this study.

**Figure 1:**
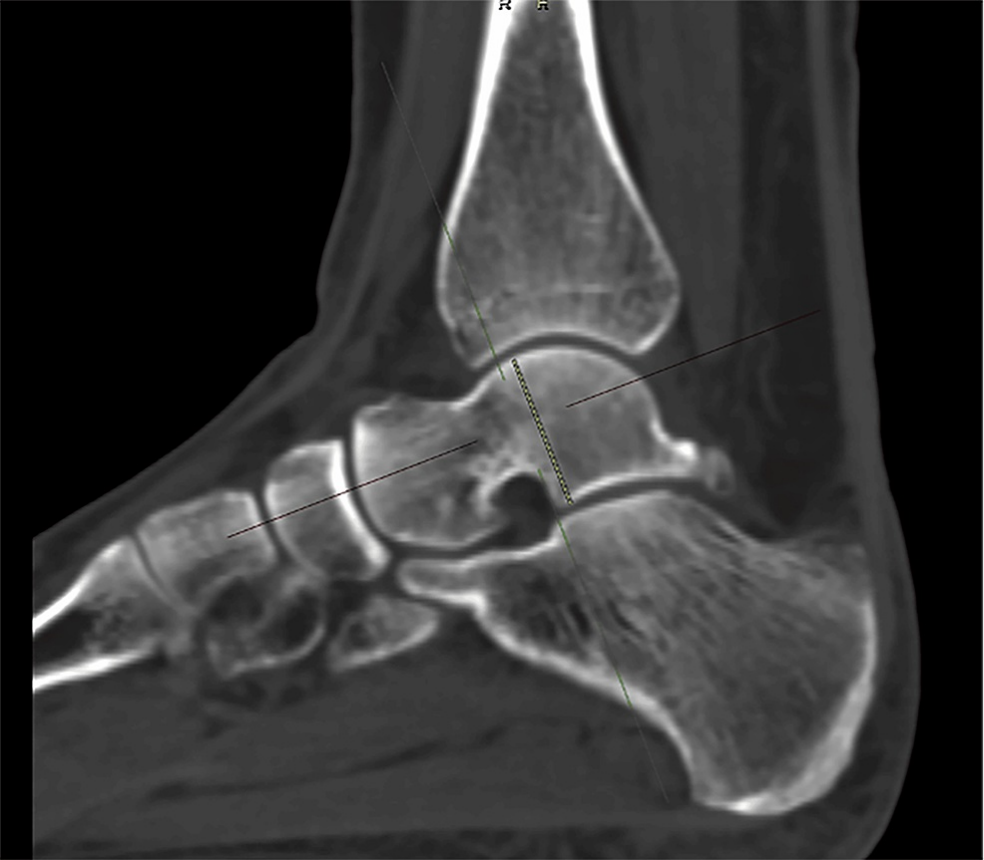
Talar body height measurement The yellow line indicates the measurement.

**Figure 2:**
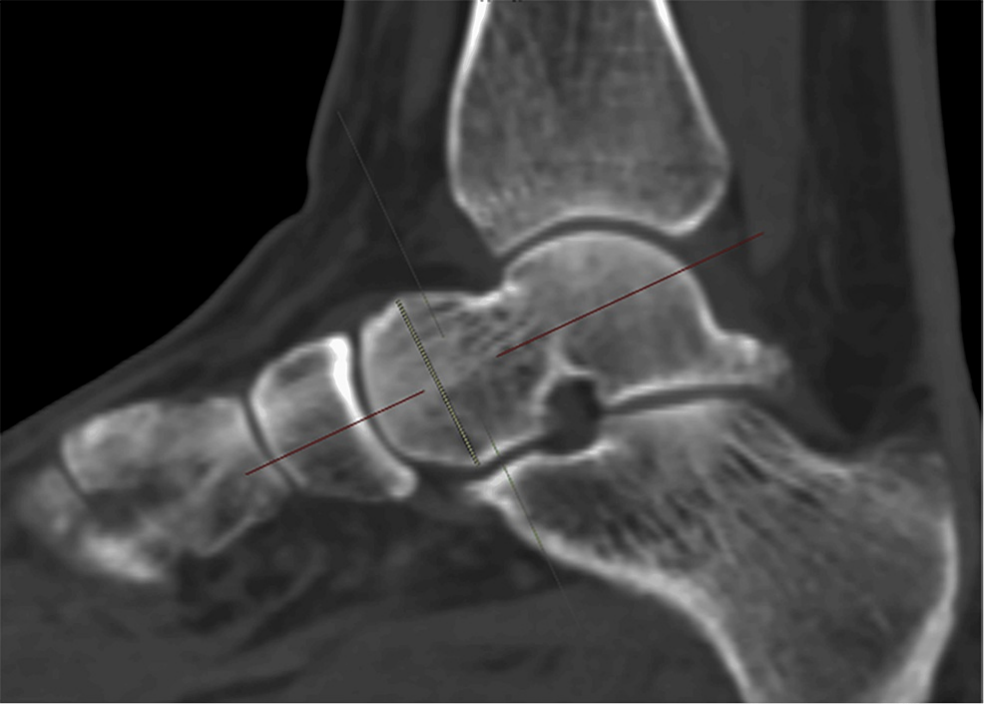
Talar head height (THH)

**Figure 3:**
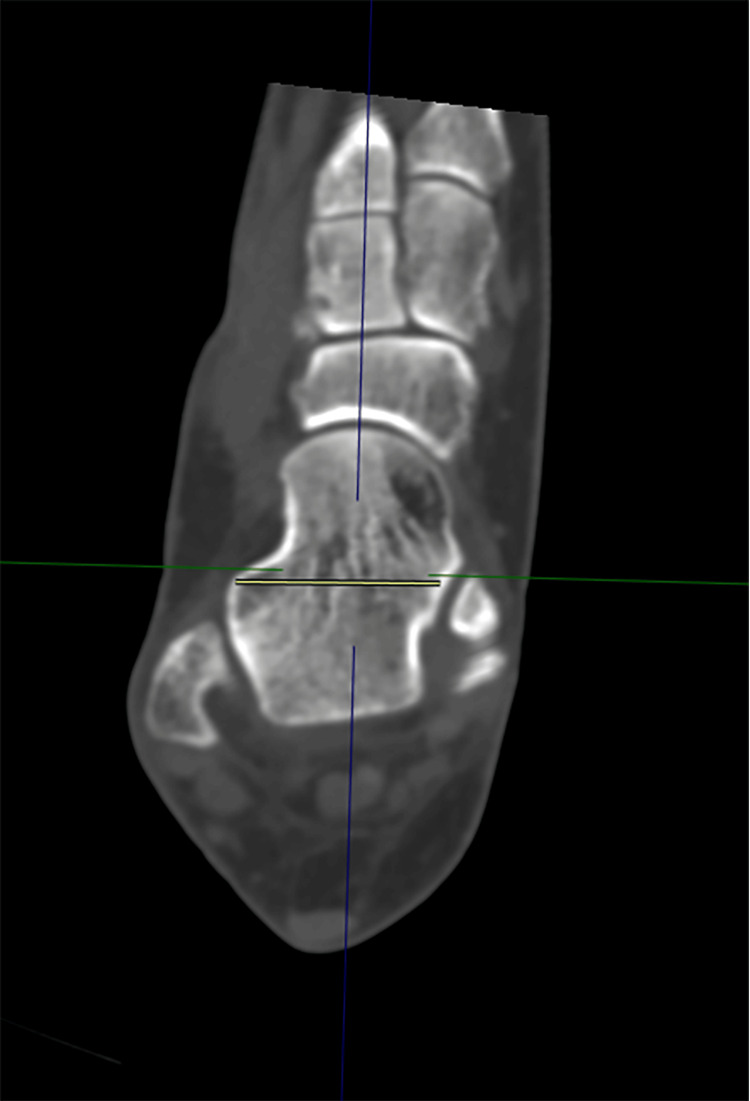
Talar neck width (TNW)

**Figure 4:**
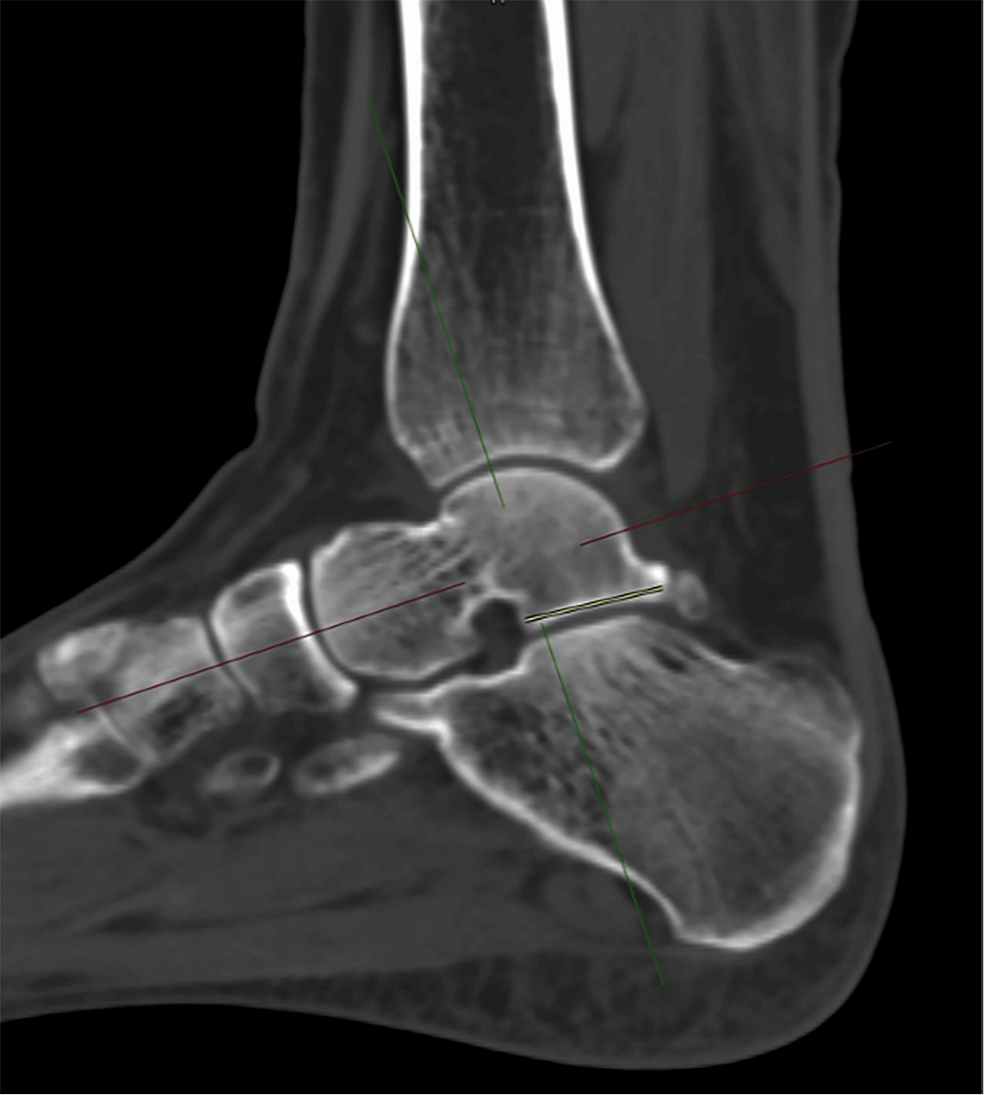
Lateral process to posterior talus

**Figure 5:**
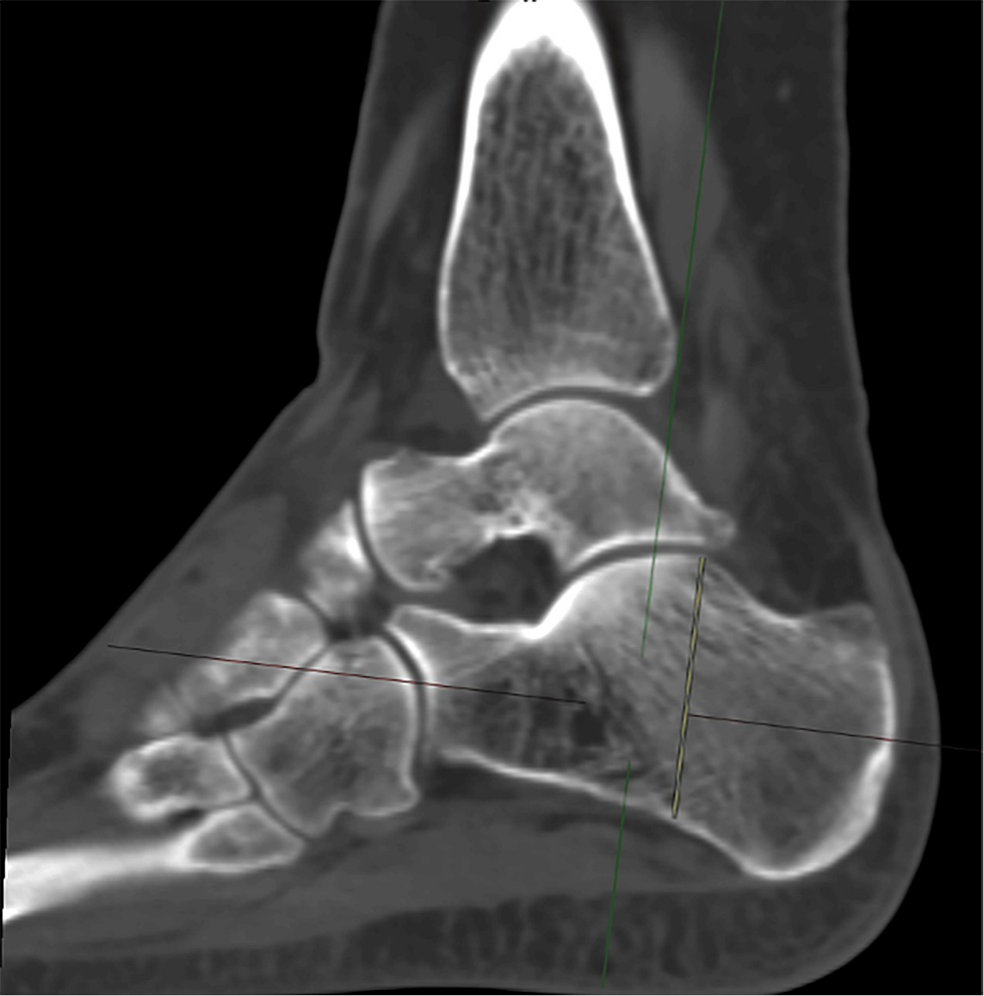
Calcaneal height (CH)

1. Talar body height (TBH): measured in the sagittal plane. The talar body midpoint was identified on axial and coronal views. Measurements were made from the apex of the talar dome, parallel to its anatomical alignment to the most inferior portion of the talar body (Figure 1).

2. Talar head height (THH): measured in the sagittal plane, with coronal and axial slices centered on the talar head. Measurement was made from the apex of the head, parallel to the longitudinal axis, to the most inferior portion of the talar head (Figure 2).

3. Talar Neck Width (TNW): measured in the axial plane, orthogonal to the anatomical axis of the neck using sagittal and coronal images to determine the midline. Measurement was made from the medial to the lateral border of the talar neck, perpendicular to its anatomical alignment through the midpoint (Figure 3).

4. Lateral process to posterior talus (LPPT): measured in the sagittal plane, with axial and coronal images used to identify the true anatomical plane and midpoint. Measured from the lateral process to the posterior talus (Figure 4).

5. Calcaneal height (CH): measured in the sagittal plane, with axial and coronal images centered on the calcaneus. The midpoint of the calcaneus was identified, in the sagittal plane. Measurements were made from the most superior aspect of the posterior facet of the calcaneus to the most inferior calcaneal point, parallel with anatomical alignment (Figure 5).

Prior to data collection, talar, and calcaneal measurements were agreed upon by the two senior authors (SRK & DB) with respect to current measurements reported in the literature. Measurements were carried out by the principal author (DOS), an orthopedic resident, after instruction from the senior author (DB), a fellowship-trained musculoskeletal radiologist with over 20 years of experience. The principal author received a formal induction on the use of the imaging software as well as detailed and stepwise instructions on how to perform all measurements. Prior to the commencement of the project, five measurements and images were saved by (DB), from which all subsequent images and measurements were compared, to ensure accuracy and reproducibility. Fifteen sets of measurements (75 measurements in total) were completed prior to the start of the project which were reviewed and discussed prior to the commencement of the study, these measurements were repeated once the authors were satisfied with the accuracy of measurements. All measurements were validated by the senior author (DB). The results were recorded in a standard Excel data sheet.

Statistical analysis

Data was collated and entered into an Excel spreadsheet program. Qualitative statistical analysis was performed using SPSS software for Windows version 22.0 (IBM Corp,. Armonk, NY, USA). Baseline descriptive statistics were calculated including mean, standard deviation, and range. Pearson’s correlation analysis was performed to identify relationships between the measurements made. A regression analysis was also performed. A power analysis was not performed due to the paucity of published literature on talar and calcaneal measurements available.

## Results

CT imaging of 124 patients was screened for inclusion in this study. Of these, 64 patients met our inclusion criteria. With respect to those excluded, 21 images contained talar or calcaneal fractures, nine had previous foot and ankle surgery two demonstrated a foot deformity such as tarsal coalition. 14 CTs did not have satisfactory image quality. Fifty-three percent (n= 34) of images measured were of the right foot.

Baseline descriptive statistics are displayed in (Table 1). The standard deviation for all talar measurements was under 3.2 mm. The CH measurement had a higher SD at 3.9 mm.

**Table 1:** Baseline descriptive statistics of talar and calcaneal measurements. Measurements in millimeters

Variable	Mean	SD	Range	Max	Min
Talar body height	27.1	2.17	9	32.2	23.2
Talar head height	25.41	2.16	11.1	32.9	21.8
Talar neck width	32.7	3.16	15.3	39.5	24.2
Lateral process to posterior talus	23.6	2.64	13.7	31.5	17.8
Calcaneal height	43.8	3.9	17.7	54	36.3

An intermediate positive correlation was found with varied strengths between measurements (P< 0.01) (Table 2). A regression analysis was also performed, however, the confidence interval failed to reach statistical significance.

**Table 2:** Pearson’s correlation coefficient between continuous variables measured.

Variable	Talar body height	Calcaneal height	Lateral process to posterior talus	Talar neck width	Talar head height
Talar body height	1.00	-	-	-	-
Calcaneal height	0.65	1.00	-	-	-
Lateral process to post talus	0.53	0.55	1.00	-	-
Talar neck width	0.68	0.59	0.60	1.00	-
Talar head height	0.69	0.55	0.39	0.49	1.00

## Discussion

The most important finding was that a low standard deviation was observed in talar measurements. These findings indicate that a novel plate design would not require an extensive range of size options. In addition, an intermediate positive correlation between measurements will support our hypothesized multiplane, multidirectional, segmental plate design in which sizing would increase uniformly for each segment.

A variety of talar and calcaneal measurement techniques using modalities other than CT [4,11-14]. As described by Peckmann et al. and further validated by Cekdemir et al., calcaneal measurements were validated measurements used in anthropometrics studies to predict sex and analyze population variability [15]. In comparison, He et al. separately described techniques to obtain measurements of the talus [4]. A number of our study metric points differ from previously published work. CH in our study is more reproducible on CT in comparison to the maximum calcaneal height (MAXH) as described by Cekdemir et al. [11].

The methodology in our study differs from published anthropometric work utilizing 3D CT reconstruction and using Vernier calipers, laser technology, and anthropometric boards [16,17]. Our study has a number of strengths including ease of access to CT scanning and reproducibility of our measurement techniques by surgeons and radiologists in clinical practice.

There are a number of limitations in this study. First, patient demographics were not included. We acknowledge a statistically significant variability in foot and ankle morphology based on gender and body mass index has been established, however, the goal of this study was to obtain measurements on a consecutive sample of patients, not to identify differences between population subgroups [4,14]. Second, inter-observer variability was not measured in this study. Senior authors agreed that a rigorous induction, multiple measurements with feedback prior to the commencement of the study, and measurements vetted by a musculoskeletal radiologist were sufficient to ensure the accuracy and reproducibility of talar and calcaneal metrics recorded. Last, our strict inclusion criteria resulted in a relatively small sample size however, our sample size is comparable to previous talar and calcaneal anthropometric and morphometric studies [4,17].

## Conclusions

Our research has expanded on previously described work on talar and calcaneal morphology. Our analysis identified a low degree of heterogeneity in measurements while also identifying through regression analysis that there is not a strong causal relationship between variables. Results indicate that extensive sizing options will not be required when selecting appropriate constructs for plate design. Our measurement techniques described may also aid foot and ankle surgeons with preoperative planning in complex cases.
